# Papular xanthomas and erosive arthritis in a 3 year old girl, is this a new MRH variant?

**DOI:** 10.1186/1546-0096-7-15

**Published:** 2009-10-08

**Authors:** Catalina Matiz, Polly J Ferguson, Andrea Zaenglein, Brandt Groh, Catherine April Bingham

**Affiliations:** 1 Division of Pediatric and Adolescent Dermatology, Rady Children's Hospital, San Diego, California, USA; 2 Department of Pediatrics, University of Iowa Carver College of Medicine, Iowa City, Iowa, USA; 3 Department of Dermatology, Penn State Milton S Hershey Medical Center, Hershey, Pennsylvania, USA; 4 Division of Pediatric Rheumatology, Penn State Milton S Hershey Medical Center, Hershey, Pennsylvania, USA

## Abstract

Xanthomatous skin lesions and arthritis in children are not a common association. We present the case of a 3 year old girl who presented with xanthomatous lesions in the periungual region of both hands, around the nares and on her forehead, associated with significant arthritis that was clinically compatible with multicentric reticulohistiocytosis. However, pathology of the xanthomatous lesions was more suggestive of papular xanthoma, a disease that is not associated with arthritis. Based on her presentation and the negative lipid workup, she was treated for presumed multicentric reticulohistiocytosis. Multiple treatment strategies were utilized, with improvement on a combination of infliximab, methotrexate, and prednisone. We review the different diagnoses that should be considered in children with xanthomas and arthritis as well as the different pharmacologic therapies used in children with multicentric reticulohistiocytosis.

## Background

Xanthomatous disorders are uncommon in childhood. The most common xanthomatous disorders seen in childhood including xanthoma disseminatum, papular xanthoma, and benign cephalic histiocytosis are not associated with arthritis. Arthritis in childhood is also relatively rare, with juvenile idiopathic arthritis accounting for the majority of cases. When xanthomatous lesions and arthritis are seen together in childhood, the differential diagnosis is limited to a relatively short list of exceedingly rare disorders which can be subdivided into two subgroups based on whether or not there are associated lipid abnormalities. When a child presents with xanthomas, arthritis, and elevated lipid levels, familial hyperlipidemia type II and sitosterolemia must be considered; when the lipid levels are normal, the differential includes Von Gierke glycogen storage disease, Farber disease, multicentric reticulohistiocytosis (MRH), and familial and histiocytic dermatoarthritis. We present a case report of a 3 year old female with a clinical picture most consistent with MRH but with pathology more suggestive of papular xanthoma.

## Case Presentation

**Figure 1:**
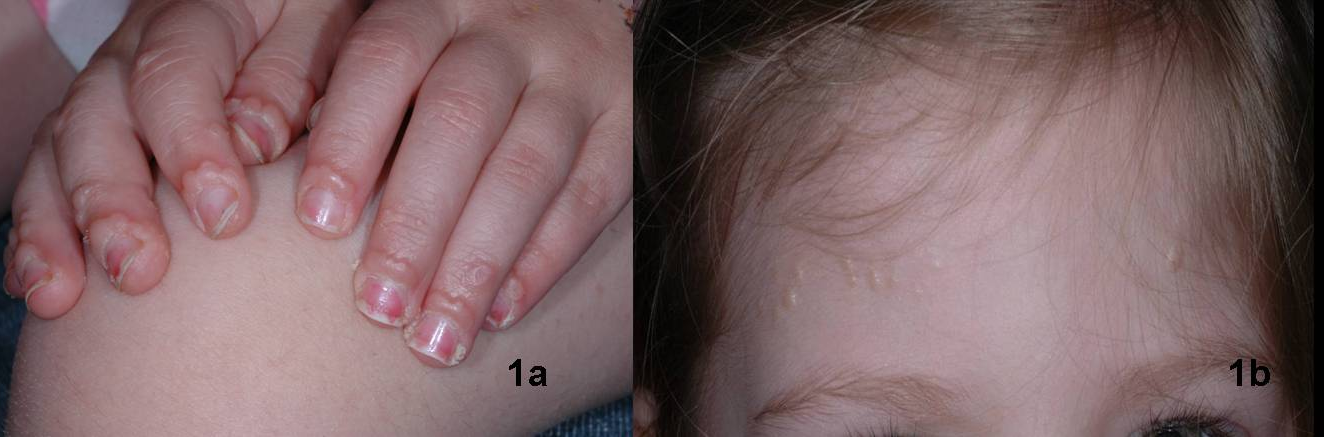
**Figures 1a and 1b.** Pink, flat topped, smooth papules overlying the proximal nail folds of the hands bilaterally **(1a)**, and on the forehead **(1b)**.

A previously healthy 3 year old girl presented to our Pediatric Dermatology Clinic in April of 2007 with a 6 month history of a papular skin eruption on the finger nail margins of her hands bilaterally, her scalp, and around the nares (fig 1). These symptoms were associated with significant arthralgia affecting her wrists and knees. There were no other associated symptoms. Her family history was negative for lipid disorders, dermatologic conditions, and chronic inflammatory conditions.

The physical examination revealed multiple flesh colored to slightly pink, flat-topped, smooth papules lined up in a row in the periungual skin on every finger of both hands, as well as the margin of the nares, forehead, and scalp. The musculoskeletal examination demonstrated unequivocal polyarthritis. There was swelling, loss of range of motion, and pain with movement in both elbows. There was synovial thickening and pain with flexion in both wrists. There were flexion contractures of the second and third proximal interphalangeal joints of both hands. Effusions and synovial thickening were present in both knees.

Laboratory results showed a normal complete blood count, hepatic profile, renal profile, and lipid profile (cholesterol 128 mg/dL, low density lipoprotein 83 mg/dL, high density lipoprotein 30 mg/dL, triglycerides 75 mg/dL). Lactate, uric acid, serum sterol, and urine organic acid levels (desmosterol, lathosterol, campesterol and sitosterol) were all within normal limits. ESR and CRP were within normal limits. Serum protein electrophoresis revealed no significant abnormalities. Lyme serology, anti-nuclear antibodies, and rheumatoid factor were negative. PPD was negative.

Bilateral hand X-rays demonstrated mild diffuse osteopenia without periosteal reaction, soft tissue swelling in the digits, most striking at the base of the fingernails and a radiolucency at the base of the left third proximal phalanx. A CT scan of the chest, abdomen and pelvis was unremarkable. CT scan of the neck showed multiple, less than 2 cm lymph nodes. A glenohumeral joint effusion was noted as well.

**Figure 2:**
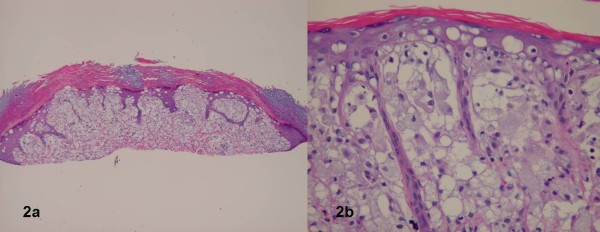
**Figures 2a and 2b.** Sections showed a dome-shape lesion with dermal infiltrate composed of foamy histiocytes.  Few admixed lymphocytes were noted.  Multinucleated giant cells and Touton type giant cells were not observed.  There was upward migration of cells with clear, foamy, and vacuolated cytoplasm with in the overlying epidermis. a) 25x, H&E staining; b) 400x, H&E staining.

Skin biopsies were obtained from the nail fold and scalp. The histology sections showed a dome-shape lesion with dermal infiltrate composed of foamy histiocytes and a few admixed lymphocytes (fig 2). Multinucleated giant cells and Touton type giant cells were not observed. There was upward migration of cells with clear, foamy, and vacuolated cytoplasm within the overlying epidermis. PAS stains were negative for glycogen deposition and fungal organisms. AFB stains were negative. Immunostaining with CD68 was positive, and CD1a stained only a few intraepidermal and rare dermal cells. Factor XIIIa stained a few scattered cells. These biopsies where read by multiple pathologists as consistent with papular xanthoma. Additional biopsies of synovium and additional skin nodule both showed foamy histiocytes with scattered lymphocytes arranged in sheets. Cytoplasm ranged from clear to bubbly to finely granular. CD68 and lysozyme stained strongly positive in histiocytes. Formal evaluation of the patient by an ophthalmologist, oncologist, and metabolic specialist revealed no additional findings.

**Figure 3:**
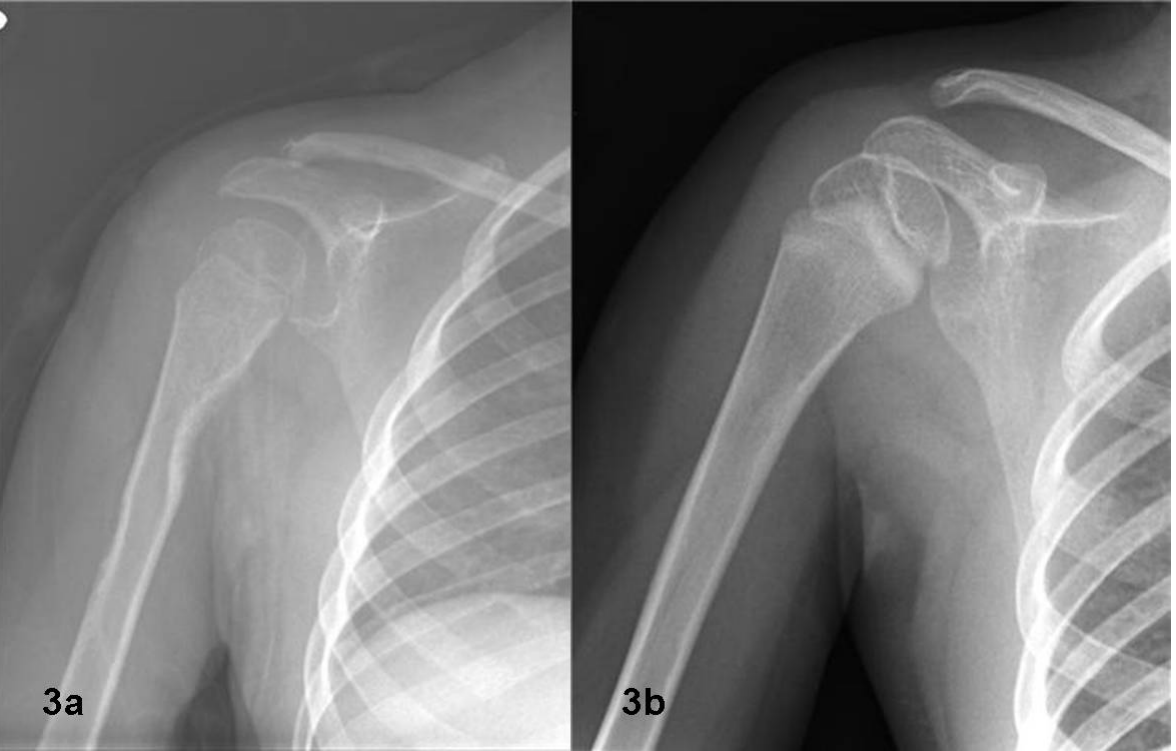
**Figures  3a and 3b.** X-ray images of the right shoulder show subtendinous resorption of bone of proximal humerus (figure 3A) followed by healing of this lesion (figure 3B) after one year of immunosuppressive medications.

The child was diagnosed with MRH based on her clinical presentation, even though histopathology was not classic for this disorder, and followed in pediatric rheumatology clinic. She was started on naproxen without major improvement in the joint complaints; methotrexate was then added at a dose of 5 mg (0.4 mg/kg) by mouth once a week and later increased to 7.5 mg (0.6 mg/kg) subcutaneously once a week without major improvement. Hydroxychloroquine 75 mg (6.25 mg/kg) by mouth per day was added. Subcutaneous nodules of about 2 × 1 × 2 cm were noted on the upper portion her arms bilaterally in the region of the axilla and diagnosed as synovial cysts by ultrasound. X ray of the shoulders revealed subtendinous erosion of bone in the humerus and distal clavicle remodeling (fig 3). Oral prednisone 1.7 mg/kg/day was started, and there was almost complete resolution of the xanthomatous lesions and moderate improvement in her arthritis. Prednisone was tapered to 1 mg/kg.

However, she continued to have multiple joint effusions and decreased range of motion in her PIP joints. Therefore, etanercept was added at a dose of 20 mg (1.25 mg/kg) subcutaneously every week. Methotrexate was increased to 12.5 mg (0.8 mg/kg). She had a partial initial response to etanercept with some improvement in the range of motion of her MCP's and wrists. However, after 3 months on etanercept, the patient had significant ongoing polyarthritis, so the etanercept dose was increased to 25 mg (1.5 mg/kg), and methotrexate was increased to 15 mg (0.9 mg/kg). As her prednisone was tapered very slowly, a few tiny xanthomatous lesions reappeared. Due to worsening polyarthritis, etanercept was discontinued, and infliximab was started at a dose of 10 mg/kg intravenously. After 2 infusions given 2 weeks apart, the patient was noted to have improvement with resolution of multiple joint effusions and improved range of motion. In addition, all xanthomas disappeared. The infliximab infusions were continued every 4 weeks. Methotrexate was continued at 0.9 mg/kg subcutaneously per week. Prednisone was tapered further, but patient developed recurrent polyarthritis and new xanthomas over her fingers. Unfortunately, her synovitis did not improve further when infliximab was increased to 20 mg/kg.

## Discussion

MRH is a systemic non-Langerhans cell histiocytosis first described in 1937 by Weber and Freudenthal [1]. Later, Goltz and Laymon coined the term multicentric reticulohistiocytosis [1]. About 200 cases have been reported since first described, but only about 10 of these cases had onset in childhood. Of these, the youngest case described is that of a 6 year old child [2].

MRH is predominantly a condition of the adult population, and it is more common in females with a ratio of about 1.85 females per 1 male [1]. MRH is characterized by severe destructive bilateral and symmetric arthritis, mainly affecting the interphalangeal joints, shoulders, wrists, and hips. Characteristic skin findings are described as cutaneous nodules or papules that are usually localized in the periungual region, pinnae of the ears, nasal folds, and scalp. The pathognomonic "coral bead sign", which our patient demonstrated, is present in about 26% of the cases [1]. Systemic findings such us fever and anemia are absent in most. An association with malignancy has been reported, but no single neoplastic disorder is directly associated with MRH. The co-occurrence of malignancy is about 15 to 25%. The reported malignancies associated with this condition include melanoma, lymphoma, breast cancer, and ovarian cancer [3,4]. MRH has also been associated with autoimmune diseases [3].

Histologic evaluation usually demonstrates multinucleated giant cells with a fine, ground-glass appearance, and in some cases, foamy or vacuolated histiocytes, as in our patient's case and that of a 14 year old girl [5,6]. Some authors report that early in the disease there are more eosinophils, lymphocytes and histiocytes with fewer number of multinucleated giant cells which increase as the disease progresses, which could explain why there were no giant cells present on our patient's biopsy [7]. There is an associated lymphocytic infiltrate. Immunohistochemistry is variable, but in most cases, staining is positive for CD68, CD4, CD45, HLA-DR, and lysozyme, but negative for S100, CD20, and factor XIIIa [7]. While the clinical presentation and course of our patient is consistent with MRH, the histopathology and age of our patient are atypical for this disorder. The biopsy was positive for CD68 and lysozyme but did not demonstrate typical MRH cells. Radiologic findings of MRH may be similar to rheumatoid arthritis, psoriatic arthritis, and osteoarthritis, but subtle differences may help to differentiate them. Radiographically, MRH presents as periarticular soft tissue swelling and juxta-articular erosions of the joints of the hands symmetrically. The erosions spread centrally and cause separation of the bony areas [8]. Osteoporosis has not been described, but osteopenia was found in our patient and was reported in a 14 year old African American girl [9]. There can be compromise of the atlantoaxial joint causing subluxation and instability. The disease can progress to arthritis mutilans [8].

The differential diagnosis of xanthomas and arthritis is short but includes familial hypercholesterolemia type II and sitosterolemia. Familial Hypercholesterolemia type II is a common, autosomal dominant genetic disorder caused by a mutation in the LDL receptor gene that may present with xanthomatous lesions and oligoarthritis. Sitosterolemia is a rare, autosomal recessive lipid disorder characterized by extremely elevated plasma plant sterol levels. It is clinically characterized by tuberous skin and tendon xanthomatous lesions, arthritis, and premature vascular disease. Our patient had normal lipid levels and sterol screening which ruled out these diagnoses.

Other disorders like Von Gierke glycogen storage disease and Farber's disease can present with xanthomas and joint complaints. The lack of systemic involvement and progressive course did not support these diagnoses in our patient. The lack of osteocondrodystrophy, ocular findings, and family history in our patient excluded familial histiocytic dermatoarthritis and dermochondrocorneal dystrophy as possible diagnoses. Xanthoma disseminatum, also part of the non-Langerhans histiocytic disorder spectrum, typically occurs in teenagers and young adults. The skin lesions are described as yellow to brown growths forming plaques and nodules that can present anywhere on the skin but most commonly in flexural areas of the extremities, the eyelids, and mucosae; visceral involvement has been described. Case reports of skeletal involvement presenting with limb pain caused by bone infiltration of xanthomas exist, but no cases of joint infiltration or associated arthritis have been published. Other non- LCH disorders such as juvenile xanthogranuloma (JXG) and generalized eruptive histiocytomas (GEH) can present with xanthomatous lesions. Skin biopsy of these lesions shows a characteristic vacuolated histiocytic infiltrate which contains Touton giant cells in 85% of the cases and a characteristic positivity of the lesions to factor XIIIa, CD68, CD163, fascin, and CD14 but negative S100 and CD1 on immunohistochemistry [10]. The histologic findings of JXG and GEH are different from those of our patient. Moreover, these entities are not known to be associated with arthritis.

Despite the clinical picture being consistent with MRH, the histologic examination of the skin lesions and the synovium were consistent with a diagnosis of papular xanthoma (PX). This entity, a normo-lipemic, non-Langerhans cell histiocytosis, is also a very rare disease in childhood. The cases reported in children have been characterized clinically by the presence of generalized yellow to brown papules and papulonodules on the face, trunk and limbs and a distinctive resolution in 1 to 5 years leaving anatoderma like scars in 60% of the case [11]. Histologic examination of the lesions shows similar characteristics as the ones seen in our patient: a dermal infiltrate of foamy histiocytes; however, our patient did not have Touton giant cells that are commonly be seen in PX. In popular xanthoma, histiocytes are CD68, KiM1p, and HAM 56 positive but negative for XVIIIa, S-100, lysozyme and CD56 [11]. Usually, these patients have no systemic involvement, and there is no report in the literature of an association with arthritis.

Multiple treatment options have been utilized in patients with MRH including NSAIDs, cyclophosphamide, azathioprine, methotrexate, hydroxychloroquine, corticosteroids and, recently, biologics with no definitive treatment regimen that can control the disease in all cases [12-14]. Several case reports in children have described acceptable results with NSAIDs or methotrexate alone [2,6]. For severe cases, more aggressive therapy is warranted. Systemic steroids can be effective for the arthritis, serositis, and xanthomatous skin lesions as was seen in our patient, but this is not a good long term treatment option given the toxicity.

The tumor necrosis factor (TNF) antagonists are a logical consideration given the evidence of increased TNF-α in patients with MRH [15]. Etanercept, an α-TNF receptor fusion protein, has been reported efficacious in some adult patients with difficult to treat MRH, but has not been successful in other reports [16]; the use of etanercept has not been reported in children with MRH. Etanercept did not completely control the synovitis in our patient. Infliximab, a monoclonal antibody against human-TNF-α, has been used in combination with methotrexate and prednisolone, with success in two case reports [12,17]; however, no benefit was achieved with infliximab in combination with methotrexate in another patient [13]. Adalimumab, a recombinant human IgG1 monoclonal antibody against TNF-α, has also been reported to be useful in the treatment of MRH [14].

There have also been reports of improved disease control when bisphosphonates are either added to or used as monotherapy to the treatment regimen in MRH [18]. Multinucleated cells from cutaneous nodules in MRH have been found to stain strongly with osteoclast markers, suggesting osteoclast differentiation [18]. In MRH, RANKL and macrophage colony stimulating factor stimulate synovial fluid macrophages to differentiate into osteoclasts [18]. Bisphosphonate therapy can decrease RANKL levels and promote osteoclast apoptosis. Therefore, bisphosphonates could be a promising treatment option for children with MRH, but more studies need to be conducted.

Preventing erosive joint destruction, particularly blocking the progression of MRH to arthritis mutilans, is the ultimate goal in treatment, and the search for an ideal treatment regimen continues. Corticosteroids seem to be very effective, but side effects limit its long term use especially in growing children. TNF inhibitors may be of benefit to some, although they were not very effective in our patient in that the effects were not sustained, and the use of TNF inhibitors did not have a steroid sparing effect. Combination regimens of several different immunosuppressive drugs seem to be the most effective way to control the disease. Bisphosphonates are another unexplored treatment option to consider in children with MRH.

## Abbreviations

MRH: Multicentric Reticulohistiocytosis; CT: computed tomography; NSAID: non: steroidal anti: inflammatory drug; MCP's: metacarpophalangeal joints; PIP: proximal interphalangeal.

## Consent

Written informed consent was obtained from the patient for publication of this case report and any accompanying images. A copy of the written consent is available for review by the Editor-in-Chief of this journal.

## Competing interests

The authors declare that they have no competing interests.

## Authors' contributions

CAB, PJF and AZ are the treating and consulting physicians. CM prepared the manuscript. CAB, PJF, AZ, BG reviewed and edited the manuscript.
